# Attention neuroenhancement through tDCS or neurofeedback: a randomized, single-blind, controlled trial

**DOI:** 10.1038/s41598-022-22245-6

**Published:** 2022-10-20

**Authors:** Gabriel Gaudencio Rêgo, Óscar F. Gonçalves, Paulo Sérgio Boggio

**Affiliations:** 1 Social and Cognitive Neuroscience Laboratory and Developmental Disorders Program, Center for Health and Biological Sciences, Mackenzie Presbyterian University, Rua Piauí, 181, 10ºandar, São Paulo, SP 01241-001 Brazil; 2 Proaction Lab, CINEICC-Faculty of Psychology and Educational Sciences, University of Coimbra, Coimbra, Portugal

**Keywords:** Placebo effect, Attention, Electroencephalography - EEG

## Abstract

Neurofeedback and transcranial Direct Current Stimulation (tDCS) are promising techniques for neuroenhancement of attentional performance. As far as we know no study compared both techniques on attentional performance in healthy participants. We compared tDCS and neurofeedback in a randomized, single-blind, controlled experiment assessing both behavioral (accuracy and time reaction) and electrophysiological (N1, P1, and P3 components) data of participants responding to the Attention Network Task (ANT). Eighty volunteers volunteered for this study. We adopted standard protocols for both techniques, i.e., a Sensorimotor Rhythm (SMR) protocol for neurofeedback and the right DLPFC anodal stimulation for tDCS, applied over nine sessions (two weeks). We did not find significant differences between treatment groups on ANT, neither at the behavioral nor at the electrophysiological levels. However, we found that participants from both neuromodulation groups, irrespective of if active or sham, reported attentional improvements in response to the treatment on a subjective scale. Our study adds another null result to the neuromodulation literature, showing that neurofeedback and tDCS effects are more complex than previously suggested and associated with placebo effect. More studies in neuroenhancement literature are necessary to fully comprehend neuromodulation mechanisms.

## Introduction

Neuroenhancement can be defined as the procedure for improving cognitive performance in healthy subjects using non-invasive methods, such as pharmacological^1^ or non-invasive brain stimulation (NIBS) techniques. Along with the spread of pharmacological stimulants^2^, there is an increase in the use of neuromodulation devices such as neurofeedback^3^ and transcranial direct current stimulation (tDCS) for neuroenhancement^4^. Given its importance to daily life, academic and professional performance, attention has been a frequent target for neuroenhancement techniques^5,6^.

Neurofeedback is a technique where one aims to modulate the brain’s activity through a feedback signal derived from this ongoing activity provided by neuroimaging (e.g., functional magnetic resonance imaging) or electrophysiology (electro- or magnetoencephalography) techniques^7^. Neurofeedback studies in attention usually targeted theta, theta/beta ratio, sensory-motor rhythm (SMR), and gamma-band modulation^8,9^. While some reviews reported improvements in attentional performance^8^, other reviews indicated null effects^9^. Given the contradictory findings, a relevant issue in neurofeedback research is to disentangle the contribution of the placebo effect in positive findings^10–12^.

In tDCS, a low-intensity current (usually between 1 and 2 mA) is generated by a stimulator and applied through electrodes placed on the scalp, which modulates cortical excitability in brain areas beneath the electrodes and can modulate cognitive and behavioral performance. Two comprehensive reviews consistently showed enhancement of attentional performance in healthy subjects after tDCS over the dorsolateral prefrontal (DLPFC) or posterior parietal cortex (PPC)^13,14^. However, there is also controversy in tDCS findings, with some studies reporting positive effects^15,16^, while others found null^16,17^ or negative results (e.g., sustainable attention impairment of participants receiving tDCS while under high cognitive load)^18^. With mention that studies researching tDCS in other cognitive functions found that its effect is dependent on several parameters such as the participants’ performance at baseline^19,20^; cognitive load during stimulation^18^; or differences in positioning, orientation, and size of tDCS electrodes^21^.

To our knowledge, no study has yet compared both neurofeedback and tDCS on attentional performance in healthy participants. Thus, it would be relevant to research the use of standard protocols of both techniques in healthy samples to test their differential effects when compared to placebo on the Attentional Network Task (ANT). ANT is a well-validated paradigm to assess the efficiency of visual attentional components of alert, orienting, and executive control^22^. Additionally, it is also compatible with EEG, which allows the correlation of attentional electrophysiological components with behavioral measures such as accuracy and time reaction. There are several Event-related Potential (ERP) studies with ANT, typically focusing on attentional components such as N1, P1, and P3^22–28^.

In this study, we aimed to research the effects of a two-week daily protocol of active and sham neurofeedback versus tDCS in healthy participants’ attentional performance. We ran a randomized, single-blind, controlled experiment to assess behaviorally (accuracy and time reaction) and electrophysiological (N1, P1, and P3 components) changes in response to the treatment. We decided on a two-week protocol with nine sessions based on neurofeedback studies on attention performance showing significant findings with ten sessions, one^8^ or two^29^ by week. We equalized number of sessions for tDCS to avoid any indirect effect associated with experiment duration between techniques. Standard protocols were adopted for both techniques: an SMR protocol for neurofeedback and the right DLPFC anodal stimulation for tDCS. Despite the existence of studies with anodal tDCS over left DLPFC, we choose to focus on right DLPFC based on previous studies pointing out its importance with phasic components of attention^30^, attentional focus^31,32^ and sustained attention^33,34^. Besides, meditation training usually leads to increased right DLPFC activity and greater attentional efficiency^31,35^. An SMR neurofeedback protocol was selected based on previous findings indicating that enhanced SMR (alone or associated with other protocols) improved attention in both healthy and clinical participants^9,29,36^.

Based on the studies previously presented, we believe that, regarding the behavioral and electrophysiological effects on the attentional task, neurofeedback will perform similarly to the sham neurofeedback and sham tDCS. In contrast, active tDCS will lead to attentional improvement compared to all other treatments. The attentional improvement detected will be expressed as a shorter reaction time and greater accuracy in the ANT and an enhanced ERP amplitude for attentional components (N1, P1, P3) in response to detecting the cues and targets on this task.

## Method

### Participants

Eighty healthy undergraduate students from Mackenzie Presbyterian University in São Paulo, Brazil, were recruited and randomly arranged in one of the four experimental groups: (1) active neurofeedback; (2) placebo neurofeedback; (3) active tDCS and (4) sham tDCS, with twenty participants each. We decided on twenty participants by group based on the literature indicating positive findings with tDCS or neurofeedback on attention performance of healthy participants with smaller sample sizes^8,15,29,37^. Following the criteria inclusion, the participants were between 18 and 35 years old, with normal or corrected-to-normal vision, and were right-handed (Edinburgh Handedness Inventory score > 80). Concerning the exclusion criteria, none of the participants reported a history of neurological or psychiatric disorder, metallic implants in the head, or were under the effect of psychoactive medication or drugs during the experiment. The study was approved by the Mackenzie Presbyterian University’s ethics committee and by the National Ethics Committee (SISNEP, Brazil; CAAE: 49534415.7.0000.0084) and was conducted according to the Declaration of Helsinki. All participants signed an informed consent and were informed that they could withdraw from the experiment at any moment.

### Instruments

*Edinburgh Handedness Inventory (EHI)*^38^—It is a ten-item questionnaire, each describing a manual activity (e.g., writing, drawing, using scissors, sweeping the house) where the participant should report their hand preference. A final quotient hanging from − 100 (left-handed) to 100 (right-handed) indicates laterality.

**Figure 1:**
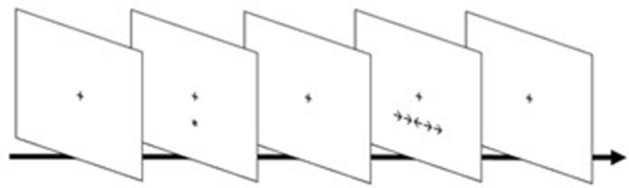
ANT trial sequence showing the screens order, with spatial cue and incongruent target as examples.

*Attention Network Task (ANT)*—We used an adapted version of ANT^39^ programmed on E-Prime to evaluate attentional performance before and after neuromodulation treatments. Participants had to look at a fixation cross in the center of a white screen and respond to a target’s appearance, i.e., a group of five arrows above or below the fixation cross, indicating the central arrow’s direction (left or right) in a response box. The central arrow could be flanked by traces (neutral condition), same direction arrows (congruent condition), or opposite direction arrows (incongruent condition). In some trials an asterisk (cue) appeared, indicating where or when the arrows would appear. The cue could flash above or below the fixation cross, indicating that target would appear soon in that place (spatial condition); or it could flash below and above the fixing cross, indicating that the target would appear soon (double condition), or cue could not appear (absent condition). The classical ANT version adopts a fourth cue condition, the center cue, however, we decided to discard this condition for two reasons: (1) we preferred not to assess attentional network scores, but the raw accuracy and reaction time for each target and cue conditions, given some studies questioning the reliability of those scores^40,41^; and (2) it allowed us to increase the trial number of the other cues, enhancing its signal-to-noise ratio and making the task shorter to the participants, avoiding their fatigue. The test consisted of 216 trials, divided into six blocks of 36 trials, each block with four repetitions of target and cue conditions (3 targets × 3 cues × 4 rep.). The sequence of stimulus in a trial, showed in Fig. 1, consisted of the following screens: (1) fixation screen, with a black fixation cross in the center of a white window (random duration between 400 and 1600 ms); (2) cue screen, with double, spatial, or absent cue conditions (100 ms); (3) a second fixation screen (400 ms); (4) target screen, where arrows appeared, and the participant should respond (duration equal to response time and a maximum time of 1700 ms); (5) a final fixation screen, which lasted until the trial duration totalized 3500 ms.

*Perceived Changes in Attentional Performance Scale (PCAPS)*—Each participant reported at the end of the treatment how much they believe their attentional performance changed in response to the treatment, on a scale of “0” (“much worse”) to “10” (“much better”), where “5” was equivalent to no perceived changes. They should answer any perceived changes in their attention to the ANT task and in their day-to-day activities, such as improved attention in work or classes in the university.

### Equipment

*Neurofeedback*—Neurofeedback intervention was conducted using a NeXus-4 EEG apparatus (Mindmedia Neuro and Biofeedback systems, Maasbracht, The Netherlands). For signal processing and neurofeedback training, we adopted the software Biotrace + from the same company. Participants did the neurofeedback protocol in a quiet room with a computer connected to two monitors, one for the researcher and a second monitor for the participant. A monopolar assembly was used for active and placebo protocols, with the active electrode placed on the vertex (Cz), the ground electrode placed on the right ear lobe (A2), and the reference electrode placed on the left earlobe (A1). The signal was corrected in the Biotrace + using the Automatic Artifact Rejection option, which rejected all the total power above 100μv and electromyography (EMG) artifacts (75–100 Hz) with power above 10μv. During the SMR protocol, the monitor presented to the participants presented several visual features: a bar graph reporting SMR amplitude; an image of a circle (similar to a mandala) that changed size according to SMR amplitude; and a bar chart showing the amplitude of EMG artifacts. Placebo neurofeedback followed the same protocol as the active, but the session exhibited to the participants in the monitor was a demonstration session, which were obtained from real EEG recordings, in this way identical to a real session. For each session, the participant did a baseline measure of SMR amplitude for 2 min. After this measure, participants performed the 20 min session, aiming to enhance SMR amplitude. During the session, participants were instructed to try to enhance their SMR activity by any means, while not enhancing the EMG signal (which indicated artifacts), and in case they reached the goal (increase SMR signal beyond 10% of the baseline) they would receive a visual reward, where the image on the screen would start moving. After each session, participants should report the occurrence of adverse effects during the session.

**Figure 2:**
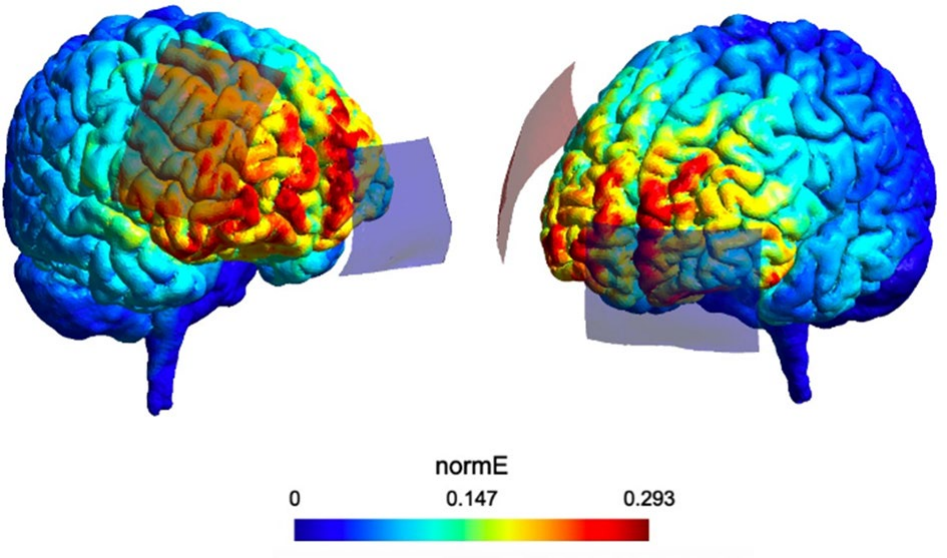
Computational model (SimNIBS software) of electrical current dispersion in cortical surface based on the tDCS montage adopted in the study. normE—Electrical field norm in volt per meter (V/m).

*Transcranial Direct Current Stimulation (tDCS)*—We applied the direct electrical current through rubber electrodes inside saline-soaked sponges fixed in the scalp by rubber bands, with the current delivered by a battery-driven stimulator. The tDCS stimulator was developed in our laboratory (São Paulo, Brazil; any technical aspect please contact psboggio@gmail,com). Anodal electrode was positioned over F4, according to the 10–20 positioning system, targeting the right DLPFC; and the reference electrode over the contralateral supraorbital region. The rubber electrodes were 35 cm2 (7 × 5 cm), and the stimulation intensity was 1.5 mA. Although most studies on this topic used 2.0 mA^8,15^, we decided to use 1.5 mA seeking greater comfort for the participant and reducing the total charge due to multiple sessions in the week. The safety aspects of the tDCS application followed the safety protocols suggested in the literature^42–44^. The sham tDCS followed the same protocol of the active, with the difference that the participant received the electric charge in the beginning and at the end of each session for only 30–15 s to ramp up to 1.5 mA and 15 s to ramp down. During the sham condition, the tDCS device showed the intensity (mA) in the display identical to the active condition to make it more believable. tDCS sessions lasted 20 min, and after each session, participants should report any side effects. We assessed the electrical current dispersion of the DLPFC montage through computational modeling on the SimNIBS, as shown in Fig. 2.

*Electroencephalography (EEG)*—To collect the electrophysiological data, we used an EEG system (Electrical Geodesics Inc., Amsterdam, The Netherlands), consisting of a Macintosh computer for signal registration, an EEG amplifier (EEG system 300), an articulated arm to hold the cap and Hydrocel caps of 128 channels of three different sizes. Each EEG electrode is connected to the scalp by a sponge, wetted with a mixture of warm water, neutral shampoo, and potassium chloride to maintain the impedance below 50 kilohms. The impedance was checked at least twice in the ANT, during the intervals between the blocks.

### Procedure

Participants were recruited via the internet and on the university campus, and those who showed interest were later contacted by email with detailed information about the experiment objectives, procedure, and inclusion/exclusion criteria. We randomly allocated the selected participants to one of the four experimental groups, and we scheduled the sessions for each one. The experiment lasted two weeks (ten days): (1) the first day with the ANT with EEG recording, and with the first 20-min neuromodulation session accordingly to their experimental group; (2) 8 days with 20-min treatment sessions (with an interval on the weekend); and (3) the tenth day with participants’ assessment in the ANT with EEG recording and the PCAPS. All the sessions occurred in Mackenzie's Social and Cognitive Neuroscience laboratory. The treatment sessions (tDCS or neurofeedback, active or sham) took place in a quiet room, with the participant at rest (for active or sham tDCS) or performing the neurofeedback task (for active or sham neurofeedback).

### EEG data processing

**Figure 3:**
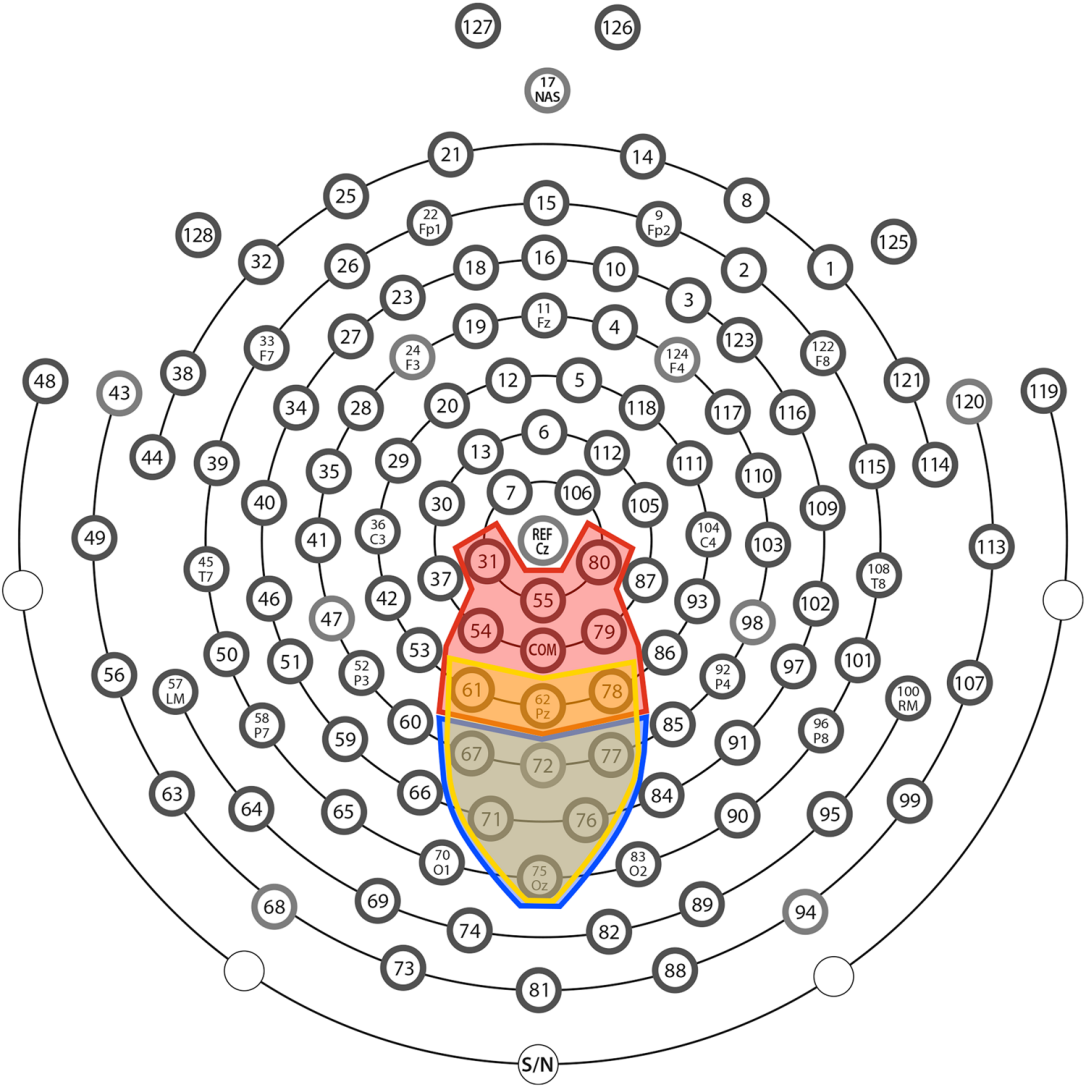
Figure depicting the top view of a 128-electrode Hydrocel geodesic cap (nose pointing up), indicating the selected cluster of electrodes to extract the P1, N1, and P3cue (in yellow); P3 parietal area (in red) and P3 occipital area (in blue).

Concerning the EEG data collected during ANT, we did a pre-processing with the following steps for ERP analysis: (1) data filtered by a 40 Hz low-pass filter; (2) segmentation of the data in − 100 to 500 ms relative to the start of each trial; (3) segments were averaged based on the type of cue and target, totaling nine categories (3 cues × 3 targets); (4) detection and marking of eye blink and movement artifact and noise (signal amplitude higher than 200 μV, blinking artifact with signal higher than 140 μV, eye movements artifact with signal higher than 80 μV); (5) exclusion of channels with more than 20% of noise and segments with more than ten noisy channels (from a total of 128), and with blink or eye movement artifacts. We inspected participants' EEG data to ascertain the number of good segments for each condition (9 conditions: 3 cues × 3 targets). Participants with losses greater than 30% (less than 50 good segments in each condition) in more than three categories (in a total of nine) were excluded. After pre-processing, we selected the time window and electrodes for the ERPs N1, P1, and P3, based on visual inspection. All the ERP were selected after target appearance, based on cue type (N1, P1, P3cue) or target type (P3). The electrodes and time window are the following: (1) P1 between 90 and 160 ms on parieto-occipital electrodes (geodesic net 61,62,78,77,67,71,76,75,72); (2) N1 between 160 and 220 ms on the same electrodes of P1; (3) P3cue—between 250 and 400 ms on the same electrodes of P1; (4) P3—between 300 and 450 ms on parietal (61,62,78,79,54,55,31,80) and occipital (71,75,76,72,67,77) electrodes. The electrode area for each ERP can be seen in Fig. 3. For each ERP component and participant, the peak amplitude was detected as the highest amplitude (positive or negative) inside the selected time window, and we extracted the mean amplitude of the peak considering a short time window around it (10 ms before and after).

### Statistical analysis

We assessed behavioral and electrophysiological data of the ANT as the primary outcomes through a mixed analysis of variance (ANOVA) with the cue (absent, double, and spatial), target (neutral, congruent, and incongruent), and session (pre- vs. post-treatment ANT) as within-subject factors; and treatment (tDCS active, tDCS sham, Neurofeedback active, and Neurofeedback sham) as between-subject factor. For the behavioral data, we analyzed accuracy (ACC), i.e., the percentage of correct answers, and the averaged reaction time (RT) given cue or target condition, which was calculated only with the correct answers in the ANT. For the ERP components, we compared the averaged amplitude in microvolts (μV) for P1, N1 P3cue, and P3. For the P3 component, we also adopted region (parietal or occipital) as a within-subject factor, to identify differences between parietal and occipital electrodes in its amplitude. We also assessed perceived changes in attention performance for each treatment group employing a one-sample t-test comparing the averaged PCAPS score against 5 (i.e., no perceived changes in attention). We also compared PCAPS scores between treatment groups through a one-way ANOVA. Finally, we assessed if any electrophysiological component would be related to behavioral performance in ANT. For this analysis, we ran Pearson correlation tests between P1, N1, P3cue, and P3 amplitudes with the averaged reaction time in ANT, with separate analyses for each session (pre-and post-treatment). For all the tests mentioned above, we adopted Tukey post hoc tests when necessary, and we adopted a statistical significance of 5%. We ran all the statistical analysis on Jamovi software (Version 1.6).

## Results

Statistical analyses were conducted with 73 participants (38 males, 35 females; 18–32 years old; mean age 23 ± 2.12). Of the total of 80 participants, four discontinued the treatment claiming conflicting schedules. One participant was excluded due to outlier data, with reaction time averages above three standard deviations in the ANT. Also, two participants were excluded due to noisy EEG data (few good segments in more than three categories, see *data processing and statistical analysis*). Participants reported no adverse effects in response to tDCS or neurofeedback and were naive to both techniques. Regarding the Neurofeedback sessions, we considered a session a success when the participant reached the goal of increasing the average SMR signal obtained by 10% beyond a baseline measure (obtained in the beginning of the same session). From the 19 participants in the active group: six participants have no success on increasing SMR in any session, seven participants have succeeded between one and three sessions; four participants succeeded between four and six sessions; and only one participant successfully increased SMR on more than six sessions, succeeding on all the nine sessions. The average number of successful sessions was 2.42 (SD: 2.47 sessions).

### Behavioral data in ANT

Regarding accuracy, we detected ceiling effects for congruent and neutral targets, with accuracy above 98% and no variance in a few cases, and for incongruent targets, the overall accuracy was higher than 90%. On average, errors in accuracy were 2.61% for the pre-treatment session (where 3.16% of errors were due to omission) and 2.54% for the post-treatment session (where 8% of errors were due to omission). Given the large skewness and the heteroscedasticity of the accuracy data when split in *cue* and *target* conditions, we analyzed only the effects of *treatment*, *session* and treatment*session on the overall accuracy (Levene’s test *p* > 0.05), showing no significant differences for any factor: *treatment* [*F*(3,69) = 0.08, *p* = 0.97], *session* [*F*(1,69) = 0.08, *p* = 0.77], nor treatment*session [*F*(3,69) = 0.73, *p* = 0.54].

**Figure 4:**
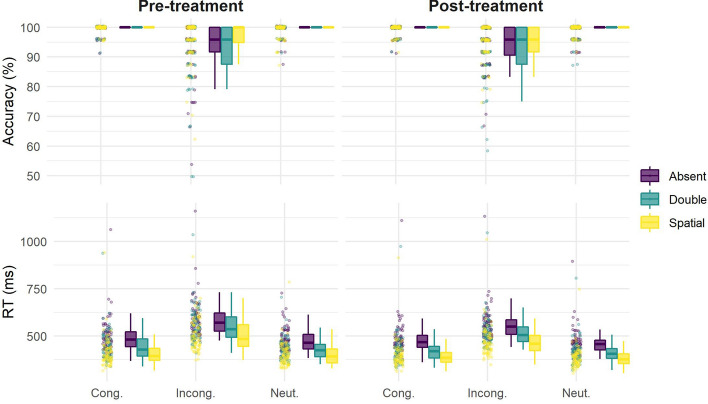
Distribution for accuracy and reaction time in Attention Network Test, showing a boxplot and point distribution of participants’ accuracy (upper part) and reaction time (bottom part) grouped by cue, target and session.

For the reaction time, we detected main effects for: *cue* [*F*(2,138) = 690.17, *p* < 0.001, *η*^*2*^*p* = 0.91], with significant differences between all conditions, where the shorter reaction time was for *spatial* and the longest for the *absent* condition; *target* [*F*(2,138) = 860.39, *p* =  < 0.001, *η*^*2*^*p* = 0.93], with significant differences between all conditions, with shorter reaction times for *neutral* and the longest for *incongruent* targets; and *session* [*F*(1,69) = 22.49, *p* =  < 0.001, *η*^*2*^*p* = 0.25], with a short reaction time in the post-treatment compared to pretreatment ANT. We also detected interaction effects between: cue*target [*F*(4,276) = 11.07, *p* = 0.14, *η*^*2*^*p* = 0.14], with significant differences between all conditions, crossing the main effects found for cue and target, as shown in Fig. 4; and target*session [*F*(2,138) = 23.57, *p* =  < 0.001, *η*^*2*^*p* = 0.25], showing differences between pre and post-treatment sessions for all target conditions, with shorter reaction time in the latter. We did not find any significant main effect or interaction associated with *treatment*, indicating no significant neuromodulation effect.

### ERP components in ANT

Concerning the P1 component, we detected main effects for *cue* [*F*(2,138) = 125.39, *p* =  < 0.001, *η*^*2*^*p* = 0.65], with significant differences between all conditions, with larger amplitude for *absent* and the smaller for *double* conditions; and *session* [*F*(1,69) = 8.54, *p* = 0.005, *η*^*2*^*p* = 0.11], with enhanced amplitude of P1 component in post-treatment ANT compared to pretreatment; but no effect for *treatment* [*F*(3,69) = 0.07, *p* = 0.98].

For the N1 component, we detected main effects for *cue* [*F*(2,138) = 172.22, *p* < 0.001, *η*^*2*^*p* = 0.71], with post hoc showing significant difference between all cue levels, but no significant differences between *sessions* or *treatments*. Post-hoc analysis showed increased N1 amplitude for double cues, diminishing spatial cues, and the smaller N1 in response to absent cues.

Regarding the P3cue, we found significant effects for *cue* [*F*(2,138) = 49.57, *p* < 0.001, *η*^*2*^*p* = 0.42], with significant differences between all conditions, with larger amplitude for absent cue and smaller for spatial cue; *session* [*F*(1,69) = 11.14, *p* = 0.001, *η*^*2*^*p* = 0.14], with enhanced amplitude for P3cue in post- compared to pretreatment ANT; and interactions between cue*session [*F*(2,138) = 5.43, *p* = 0.005, *η*^*2*^*p* = 0.07], and cue*session*treatment [*F*(6,138) = 2.16, *p* = 0.05, *η*^*2*^*p* = 0.08]. The post-hoc for cue*session showed significant difference between all cue conditions in both pre- and post-treatment sessions, similar to the main effect detected for cue (larger amplitude for absent and smaller for spatial), and a significant difference between session pre- and post-treatment only for double cues, with no differences for spatial or absent cues. However, this difference for double cues between pre- and post-treatment was detected only for sham tDCS group, as indicated by the interaction cue*session*treatment.

**Figure 5:**
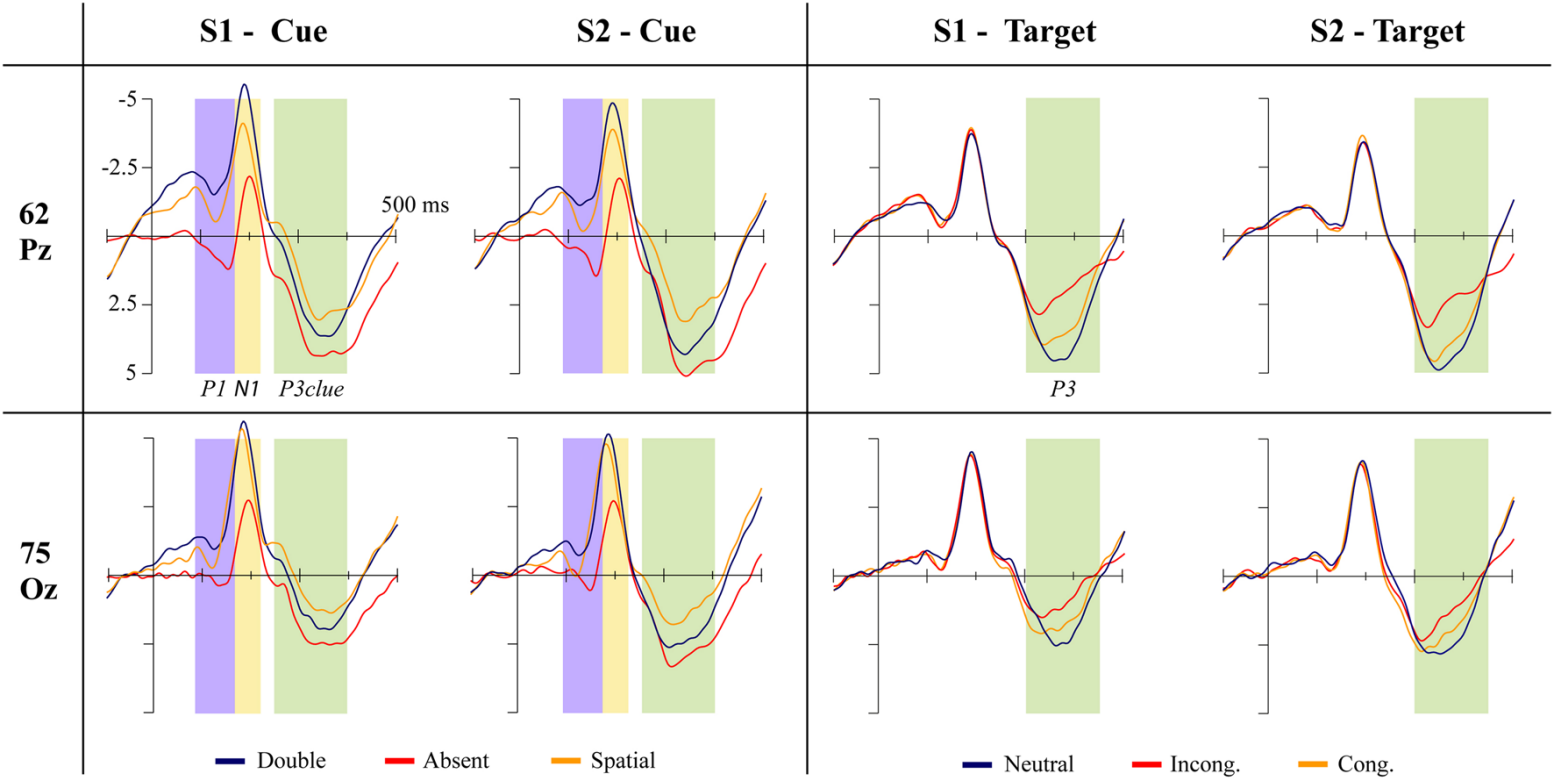
Averaged waveforms for cue and target conditions on Oz and Pz electrodes. It is grouped by session pre- or post-treatment, respectively S1 and S2. The figure shows each ERP time-window, denoted with colored squares (P1 = purple; N1 = yellow; P3cue = green; P3 = green).

Finally, the analysis for P3 component in response to targets showed main effects for *target* [*F*(2,138) = 118.91, *p* = 0.001, *η*^*2*^*p* = 0.63], with significant differences between all conditions, where neutral targets elicited the largest P3 amplitude and the incongruent the smallest amplitude; *session* [*F*(1,69) = 7.87, *p* = 0.007, *η*^*2*^*p* = 0.1], with larger P3 amplitude for post- compared to pretreatment ANT; *region* [*F*(1,69) = 6.86, *p* = 0.01, *η*^*2*^*p* = 0.09], with larger P3 amplitude for parietal compared to occipital electrodes; and the interaction target*region*treatment [*F*(6,138) = 2.2, *p* = 0.05, *η*^*2*^*p* = 0.09]. Despite this interaction reached significance level, we searched only for post-hoc comparisons of interest, i.e., differences in P3 amplitude between treatments for a same target and region, or differences between regions for a same target and treatment, but no differences were identified for those comparisons (in all cases we detected *p* > 0.1). The ERP results here presented can be visualized in Fig. 5.

### Perceived Changes in Attentional Performance Scale (PCAPS)

One sample t-tests indicated that all participants in all the four treatment groups perceived an enhancement in their attentional performance in response to the treatment, as indicated in Table 1. Despite few participants reporting no attentional improvement in response to the treatment (number of participants—active tDCS: 1; sham tDCS: 4; active neurofeedback: 3; sham neurofeedback: 3), no participant reported worsened performance. We ran an one-way ANOVA to test differences between treatment groups in PCAPS score, showing no significant differences [*F*(3,38) = 0.98, *p* = 0.41].

**Table 1:** Results for one-sample t-tests against 5 for each treatment group, with mean score, degrees of freedom (df), *t*-value and *p*-value.

Treatment	Mean score	df	*t*	*p*
Active tDCS	7.17	17	8.08	< 0.001
Sham tDCS	6.58	18	5.32	< 0.001
Active Neurof	7.15	18	9.40	< 0.001
Sham Neurof	6.88	16	6.73	< 0.001

### Correlation between ERP and reaction time

We tested for correlation between ERP (P1, N1, P3cue, and P3) and reaction time in each ANT session separately, finding a significant negative correlation between P3cue and RT, as well as P3 and RT for both sessions (pre- and post-treatment), as shown on Table 2.

**Table 2:** Pearson correlation between P3cue and P3 with RT, for pre- and post-treatment.

Pre-treatment		Post-treatment	
	RT		RT
P3cue	*r* = − 0.46; *p* < 0.001	P3cue	*r* = − 0.40; *p* < 0.001
P3	*r* = − 0.50; *p* < 0.001	P3	*r* = − 0.51, *p* < 0.001

### Complementary analyses

We ran the following complementary (post hoc) statistical analyses that were suggested during the manuscript review: (1) a mixed ANOVA with the attentional network scores (alert, orientation, and executive control) as dependent variables and with session and treatment as factors, where those scores were calculated following the method by Wang et al.^45^; (2) and a mixed ANOVA with exponential-gaussian parameters for reaction time (mu, sigma, and tau) as dependent variables and target, session, and treatment as factors; (3) ANCOVA models considering accuracy and RT as dependent variables, similar to the ANOVA models for both variables (presented on “Behavioral data in ANT”), however with perceived changes (PCAPS score) as a covariate. All analyses showed no significant main effect or interaction considering the treatment factor, which is the factor of our interest. In the supplementary information, we described in detail the results of the three analyses.

## Discussion

The present study assessed attentional neuroenhancement through two neuromodulatory techniques, tDCS, and Neurofeedback. As our primary outcome, we compared behavioral and electrophysiological performance of participants in ANT pre-and post-treatment, but we did not find significant differences given the treatment group. However, we find that participants from the four treatment groups reported, through a subjective scale, attentional improvement in response to the treatment. The lack of any effect for active treatment goes against part of the literature, which shows in some cases a positive effect of neuromodulation on healthy participants’ performance on the ANT task, both for neurofeedback^46,47^ and tDCS^15,16^.

As for the neurofeedback, we did not find any effect on the visual attentional performance of healthy participants in the ANT, contrary to some studies^46,47^. Some differences regarding the neurofeedback application may explain this distinction between our null findings and the studies that obtained positive results in ANT. For example, those studies used distinct frequency bands, modulating theta frequency or an SMR/theta protocol, aiming to increase SMR power while inhibiting theta^46,47^. Other studies that focused on improving healthy participants' attentional performance by SMR, but with other attentional tasks, also involved training other frequencies, such as beta1 or theta^48,49^. In this way, it is possible that adopting only SMR training was not so efficient compared to controlling SMR along with beta or theta frequencies. Another possible explanation for our null result is the low success rate of our participants in reaching the neurofeedback goal, where one-third failed in all sessions, and only three succeeded in more than half of the sessions. This proportion of non-respondent is in line with the literature^50–52^, indicating that a considerable part of participants (around 50%) are non-respondents (or non-learners) to neurofeedback, having no success in modulating their brain activity through this technique. Future studies must assess the neuroenhancement effectiveness of neurofeedback, focusing on participants who are respondents to the neurofeedback.

Regarding the tDCS, despite some literature showing positive results, there are also mixed findings, with studies stimulating proximal cortical areas and finding contradictory effects on attention^15,16^. For example, Miler et al.^15^ found that anodal tDCS (2 mA) over the left dorsolateral cortex enhanced executive attention compared to sham, while Roy et al.^16^ found no effect of anodal tDCS (1.5 mA) over the same area in an attentional network. Similarly, Coffman et al.^13^ found enhanced alerting effect after anodal tDCS (2 mA) over right inferior gyrus compared to sham, where Trumbo et al.^17^ detected an inverse effect, with an increased alerting effect for the sham group compared to anodal tDCS (2 mA) over right inferior gyrus. In this way, our finding is in line with Trumbo et al.^17^, showing no significant effect for active tDCS compared to sham.

Some issues could be associated with this null effect in tDCS. First, it is essential to highlight that several other tDCS studies have been finding null effects on attention^53^ and in other cognitive functions, such as working memory^54^, or physiological processes, such as substance craving^55,56^. Several studies have been discussing the contradictory effects of tDCS^57^, which could be becoming more common due to the recognition of publishing bias, such as the file drawer effect^58–60^; and also related to complex mechanisms associated with tDCS parameters that can modulate its effects, such as: dose^61,62^, participants’ baseline performance^19,20^; timing of tDCS application, i.e., if before or during a cognitive or behavioral task^63,64^; anatomical differences between subjects^65^; or even their arousal level during the neuromodulation^66^. Because of the complex effects of tDCS, future studies must investigate the interaction between these effects, identifying the individual parameters or factors that optimize the application of this technique in healthy participants. Second, several tDCS studies in the literature did not evaluate how their tDCS montage would properly stimulate the target area. To test this, we assessed if the tDCS montage we adopted (a common montage in cognitive literature) would efficiently target the DLPFC by means of SimNIBS^67^, a toolbox that models the electrical current dissipation in the brain. This modeling showed that DLPFC montage (F4 and reference over contralateral supraorbital area) is not properly targeting the DLPFC, but it is most oriented to the dorsomedial prefrontal region. Given this, we also suggest properly assessing tDCS montages using computational models or neuro-navigational methods to guarantee electrical current focus on the brain target area.

Another critical issue behind the contradictory effects in tDCS and neurofeedback is the influence of placebo effects. In our study, although we did not identify any behavioral or electrophysiological effects on ANT in response to the treatment, we detected a significant effect on the Perceived Changes in Attentional Performance Scale for all the treatment groups (active and sham), indicating that, on average, participants in all groups reported improvement in attention in response to the treatment and with a similar average score. Since sham and active groups had similar behavioral and electrophysiological performance and a similar perception of attentional improvement, it is most likely that the detected improvement was due to a placebo effect. The placebo effect in neuromodulation techniques has recently been discussed for neurofeedback^11,68^ and tDCS^69,70^, and our findings support this literature. One possibility for the placebo effect in our study is the learning effect on the task, which may have influenced participants' perception regarding their attentional improvement. We found an interaction effect between target and session, with shorter RT times for all targets in post-treatment ANT session (more expressively to the incongruent targets) and not dependent on treatment group, indicating a learning effect of participants in the task, what as previously described in the literature^71^. However, it is essential to emphasize that several participants reported that such improvements were noticeable in their daily lives, such as at work or in class performance.

Regarding the behavioral data, we found similar patterns of accuracy and reaction time to the literature: with lower accuracy for incongruent targets^22,25,28,72^, and a similar RT pattern for the target (shorter RT for neutral and congruent, longer RT for incongruent targets) and the cue (shorter RT for spatial cues, increasing for double/central cues and the longest RT for absent cues)^22,25,28,39,71,72^. As for the electrophysiological data, we detected significant effects for all ERP components concerning cues or target detection.

For the P1, we identified higher amplitudes in response to absent cues, diminishing for double cues and smaller for space cues. Thus, P1 seems to index automatic visual attentional demand level to the target surging accordingly to the informational level of the cue. This result is similar to two other studies, showing significant differences between absent and double cues, and between spatial and absent cues^26,28^. For the component N1, we found increased amplitude in response to double cues compared to absent cues, according to the literature^24,26–28^. We also found enhanced N1 amplitude for spatial compared to absent cues, which was detected by Neuhaus et al.^24^, and go in line with previous findings showing increased N1 amplitude during detection of visual stimuli in attended- compared to unattended-locations^73,74^. As for P3cue, we did not find P3 analysis in the target window given cue type in the literature. For P3pt, we identified an identical pattern to P1, with the amplitude decreasing for absent, double, and smaller for spatial cues. The hypothesis for this effect is similar to that presented for P1, i.e. P3cue indexed cognitive demand during target processing in response to the cue informational level. Regarding the P3 findings in response to the target type, we found decreased amplitude for incongruent, increasing for congruent and the largest for neutral targets, which goes in line with the literature, usually reporting decreased amplitude for incongruent due to response inhibition mechanisms after detecting incongruent targets^24–27^.

We also found learning effects for P1, P3cue, and P3 in the ANT, with greater amplitude for those components in the post-treatment session. There are no similar descriptions in the literature since we have not identified studies evaluating electrophysiological components in the ANT on different sessions. Some studies that investigate ERP in other attentional tests on two different moments detected greater amplitudes for component P3 post-intervention, usually related to enhanced attentional performance in the last moment^75,76^. Given this, a possible hypothesis is that the increased P1, P3cue, and P3 on post-treatment ANT is indexing enhanced efficiency in automatic and controlled attentional processes, which could explain the learning effect detected in the RT. It seems to be the case, at least for P3cue and P3 components, since we found a negative correlation between those components and RT. In addition, increased P3 components have been previously associated with faster RT in the literature^77–79^, similar to our findings for target conditions, where increasing P3 amplitude between conditions (incongruent, congruent and neutral) was related to decreasing RT on them.

Finally, it is essential to discuss the limitations of our study. As a first limitation, we sought to evaluate the two neuromodulation techniques based on montages suggested in the literature; however, there are other montages and parameters that we did not assess and could have enhanced attentional performance. Given this, future studies should further investigate other tDCS montages, such as targeting parietal lobe or left DLPFC; or also test other parameters, such as applying tDCS online (i.e., while the volunteer performs the attentional task). Also, our montage (F4/contralateral supraorbital area) could have facilitated current shunting^80^, despite it being suggested to happen when electrodes are 5 cm of distance or less^81^. The same could be said for neurofeedback, i.e., we recommend future studies to assess other parameters that could be more effective. such as testing quantitative EEG, dividing each session in multiple blocks to enhance learning, applying more sessions, or aiming to modulate other signals, such as SMR/theta ratio. Also, it is relevant to highlight that we decided on a small sample size in our study (twenty by group). Although other studies with tDCS^15,37^ and neurofeedback^8,29^ of attention performance in healthy subjects had significant findings with smaller sample sizes (between nine and fifteen), our sample size could have underpowered our study, given the suggested small effect size for tDCS in the recent literature^82^. Thus, our small sample size does not allow us to conclude about the effectiveness of both techniques in neuroenhancement. We suggest that future studies adopt larger sample sizes to guarantee sufficient power to the experiment. Another limitation is that we did not fully assess all the distinct attentional domains (e.g., concentration) or modalities (e.g., auditory attention) or other cognitive domains, such as memory, motivation, etc., which could have explained the improvement reported by the participants. Also, in our study we assessed only college students, who are expected to represent a specific subpopulation with cognitive performance above average^83^, and who are also young. In this way, our sample does not allow us to generalize our findings to other populations with other educational levels and ages. Finally, our study was only single-blind, which can be considered a source of bias in comparison to a double-blind study. Despite all the limitations, our study points to the relevance of possible placebo effects along with both techniques, which was previously suggested in the literature and deserves to be considered in future neuroenhancement studies.

## Conclusions

The present study endorses the literature indicating null effects of neuromodulatory techniques for neuroenhancement, suggesting the urgency for more studies in this area to understand which factors may be crucial for the effectiveness of such techniques, as well as studies properly controlling placebo effect, such as expectations related to neuromodulation or subjective perception of improving due to the treatment. In line with the literature in this topic, our study indicates how complex neuromodulation mechanisms are, and that placebo could be more relevant to its effects than previous thought. Given this, literature in this area should advertise for cautiousness^84^, mainly due to the emergence of clinics or DIY culture offering neuroenhancement as a "scientifically based" treatment with positive effects.

## Supplementary Information

Supplementary Information.

## Data Availability

The datasets generated during and/or analyzed during the current study are available from the corresponding author on reasonable request.
