# Supplementary material for: Attention neuroenhancement through tDCS or neurofeedback: a randomized, single-blind, controlled trial
Source: Sci Rep. 2022 Oct 20;12:17613. doi: 10.1038/s41598-022-22245-6 (PMC9584934; doi:10.1038/s41598-022-22245-6)
Supplement: Supplementary file 1 — Supplementary Information. [file 41598_2022_22245_MOESM1_ESM.pdf]

Title: Attention neuroenhancement through tDCS or Neurofeedback: a randomized, single-blind, controlled trial.

Authors: Gabriel Gaudencio Rêgo‡[1]; Óscar F. Gonçalves[2]; Paulo Sérgio Boggio[1].

SUPPLEMENTARY MATERIAL

Mixed ANOVA for attention network scores

We performed post hoc statistical tests (repeated measures ANOVA) comparing the scores in the three attentional networks: alerting, orienting, and executive control (flanker effect), with session and treatment as factors. There were significant main effects only for the session factor on both executive control scores (i.e., flanker effects with and without cues), as well as a statistical tendency for session factor on alerting score; however, we did not find any significant effect given the treatment factor. This result shows enhanced efficiency for the executive control network between sessions, suggesting a learning effect. Statistical data are present in table S1.

Table S1 - Mixed ANOVA results for each attention network (alerting, orienting and executive control).

Attention Network	Factor	DF	F	p	η^2p
Alerting	Session	1,69	3.83	.05	0.05
	Treatment	3,69	1.46	.23	0.06
	Session * Treatment	3,69	0.04	.99	< 0.01
Orienting	Session	1,69	2.1	.15	0.03
	Treatment		0.08	.97	< 0.01
	Session * Treatment	3,69	0.98	.41	0.04
Executive Control (without cue)	Session	1,69	4.52	.04	0.06
	Treatment	3,69	0.92	.44	0.04

	Session * Treatment	3,69	0.36	.79	0.02
Executive Control (with cue)	Session	1,69	9.87	.002	0.13
	Treatment	3,69	0.16	.92	<0.01
	Session * Treatment	3,69	0.06	.98	<0.01

Mixed ANOVA for exponential-gaussian (ex-gauss) parameters

We ran mixed ANOVA analysis with the exponential-gaussian parameters of reaction time (mu, sigma and tau). Regarding the mu parameter, we detected significant main effects for target and session, and target*session interaction. For the sigma parameter we detected significant main effects for target and session. There was no significant effect for tau parameter. All the results are described in the table S2.

Table S2 – Mixed ANOVA for each ex-gauss parameter: Mu, Sigma and Tau.

Ex-Gauss Parameter	Factor	F	Df	p	η^2p
Mu<br>Sigma	Session	32.71	1,69	< .001	0.32
	Target	427.97	2,138	< .001	0.86
	Treatment	0.23	3,69	.87	0.01
	Session*Target	3.95	2,138	.02	0.05
	Session*Treatment	0.96	3,69	.42	0.04
	Target*Treatment	1.01	6,138	.42	0.04
	Session*Target*Treatment	1.15	6,138	.34	0.05
	Session	5.88	1,69	.02	0.08

	Target	20.08	2,138	< .001	0.23
	Treatment	0.32	3,69	.81	0.01
	Session*Target	1.56	2,138	.22	0.02
	Session*Treatment	0.61	3,69	.61	0.03
	Target*Treatment	0.94	6,138	.47	0.04
	Session*Target*Treatment	1.29	6,138	.27	0.05
Tau	Session	0.02	1,69	.88	< 0.01
	Target	2.53	2,138	.08	0.04
	Treatment	0.41	3,69	.75	0.02
	Session*Target	0.71	2,138	.49	0.01
	Session*Treatment	2.12	3,69	.11	0.08
	Target*Treatment	1.45	6,138	.20	0.06
	Session*Target*Treatment	1.16	6,138	.33	0.05

Mixed ANCOVA for Reaction Time and Accuracy

We tested ANCOVAs models based on the ANOVAs adopted for RT and accuracy (as presented in the topic “behavioral data in ANT”), with the difference that we added perceived change on attention (PCAPS score) as a covariate. For RT analysis, we only found significant main effects for cue and target. For accuracy, we found significant main effects for the session and the following interactions: session*PCAPS, target*session, and target*session*PCAPS. For both analyses, there were no significant effects regarding treatment. The result of both analyses is presented in table S3.

Table S3 – Mixed ANCOVA for accuracy (ACC) and reaction time (RT).

Dependent Variable	Factor	F	Df	p	η^2p
ACC	Target	0.07	2,136	.94	<0.01
	Target*Treatment	0.06	6,136	.99	<0.01
	Target*PCAPS	1.02	2,136	.37	<0.02
	Session	9.16	1,68	.003	0.12
	Session*Treatment	1.30	3,68	.28	0.05
	Session*PCAPS	9.71	1,68	.003	0.13
	Target*Session	3.57	2,136	.03	0.05
	Target*Session*Treatment	7.51	6,136	.08	0.08
	Target*Session*PCAPS	16.64	2,136	.02	0.06
	Treatment	0.06	3,68	.98	<0.01
	PCAPS	0.53	1,68	.47	<0.01
RT	Cue	13.28	2,136	<.001	0.16
	Cue*Treatment	0.53	6,136	.79	0.02
	Cue*PCAPS	0.33	2,136	.72	<0.01
	Target	27.11	2,136	<.001	0.29
	Target*Treatment	0.21	6,136	.97	<0.01
	Target*PCAPS	0.34	2,136	.71	<0.01
	Session	1.35	1,68	.25	0.02
	Session*Treatment	1.17	3,68	.33	0.05

	Session*PCAPS	0.17	1,68	.68	<0.01
	Cue*Target	1.10	4,272	.36	0.02
	Cue*Target*Treatment	0.87	12,272	.58	0.04
	Cue*Target*PCAPS	0.48	4,272	.75	<0.01
	Cue*Session	2.33	2,136	.10	0.03
	Cue*Session*Treatment	0.90	6,136	.50	0.04
	Cue*Session*PCAPS	1.82	2,136	.17	0.03
	Target*Session	0.62	2,136	.54	<0.01
	Target*Session*Treatment	0.30	6,136	.94	0.01
	Target*Session*PCAPS	0.17	2,136	.84	<0.01
	Cue*Target*Session	1.63	4,272	.17	0.02
	Cue*Target*Session*Treatment	0.65	12,272	.80	0.03
	Cue*Target*Session*PCAPS	1.56	4,272	.19	0.02
	Treatment	0.07	3,68	.98	<0.01
	PCAPS	0.15	1,68	.70	<0.01
